# Genetic Elucidation of Human Hyperosmia to Isovaleric Acid

**DOI:** 10.1371/journal.pbio.0050284

**Published:** 2007-10-30

**Authors:** Idan Menashe, Tatjana Abaffy, Yehudit Hasin, Sivan Goshen, Vered Yahalom, Charles W Luetje, Doron Lancet

**Affiliations:** 1 Department of Molecular Genetics, Weizmann Institute of Science, Rehovot, Israel; 2 Crown Human Genome Center, Weizmann Institute of Science, Rehovot, Israel; 3 Department of Molecular and Cellular Pharmacology, Miller School of Medicine, University of Miami, Miami, Florida, United States of America; 4 Department of Otolaryngology, Meir Hospital, Kfar Saba, Israel; 5 National Blood Group Reference Laboratory, Magen David Adom National Blood Services Center, Ramat-Gan, Israel; North Carolina State University, United States of America

## Abstract

The genetic basis of odorant-specific variations in human olfactory thresholds, and in particular of enhanced odorant sensitivity (hyperosmia), remains largely unknown. Olfactory receptor (OR) segregating pseudogenes, displaying both functional and nonfunctional alleles in humans, are excellent candidates to underlie these differences in olfactory sensitivity. To explore this hypothesis, we examined the association between olfactory detection threshold phenotypes of four odorants and segregating pseudogene genotypes of 43 ORs genome-wide. A strong association signal was observed between the single nucleotide polymorphism variants in *OR11H7P* and sensitivity to the odorant isovaleric acid. This association was largely due to the low frequency of homozygous pseudogenized genotype in individuals with specific hyperosmia to this odorant, implying a possible functional role of *OR11H7P* in isovaleric acid detection. This predicted receptor–ligand functional relationship was further verified using the *Xenopus* oocyte expression system, whereby the intact allele of *OR11H7P* exhibited a response to isovaleric acid. Notably, we also uncovered another mechanism affecting general olfactory acuity that manifested as a significant inter-odorant threshold concordance, resulting in an overrepresentation of individuals who were hyperosmic to several odorants. An involvement of polymorphisms in other downstream transduction genes is one possible explanation for this observation. Thus, human hyperosmia to isovaleric acid is a complex trait, contributed to by both receptor and other mechanisms in the olfactory signaling pathway.

## Author Summary

Humans can accurately discern thousands of odors, yet there is considerable inter-individual variation in the ability to detect different odors, with individuals exhibiting low sensitivity (hyposmia), high sensitivity (hyperosmia), or even “blindness” (anosmia) to particular odors. Such differences are thought to stem from genetic differences in olfactory receptor (OR) genes, which encode proteins that initiate olfactory signaling. OR segregating pseudogenes, which have both functional and inactive alleles in the population, are excellent candidates for producing this olfactory phenotype diversity. Here, we provide evidence that a particular segregating OR gene is related to sensitivity to a sweaty odorant, isovaleric acid. We show that hypersensitivity towards this odorant is seen predominantly in individuals who carry at least one copy of the intact allele. Furthermore, we demonstrate that this hyperosmia is a complex trait, being driven by additional factors affecting general olfactory acuity. Our results highlight a functional role of segregating pseudogenes in human olfactory variability, and constitute a step towards deciphering the genetic basis of human olfactory variability.

## Abstract 3

Genetic epidemiology analysis reveals a multifaceted mechanism underlying enhanced olfactory sensitivity to the sweaty odor of isovaleric acid in humans.

## Introduction

Humans are highly variable in their olfactory perception. Such phenotypic diversity has been known for nearly a century, indicating a widespread occurrence of odorant-specific sensitivity variations [1–6] as well as of differences in general olfactory acuity [1–3]. These variations in chemosensory sensitivity often span several orders of magnitude [1,4–8], with extreme manifestation in specific anosmia or “smell blindness” [9–11]. Such human olfactory traits have been suggested to constitute a complex trait, affected by both environmental and genetic factors [6,8,11–13]. While specific anosmia and its milder representation, specific hyposmia, have been extensively studied, less attention has been devoted to the study of the other extreme of the olfactory threshold spectrum, hyperosmia [14,15].

Considerable evidence points to a significant genetic contribution to olfactory threshold variation. For example, a higher concordance of olfactory thresholds towards androstenone was found in monozygotic twins than in dizygotic twins [13,16]. Moreover, a family-based study with pentadecalactone and isovaleric acid (IVA) [11] suggested that for some odorants anosmia constitutes a recessive Mendelian trait. Corroborating evidence was reported in particular mouse strains [17], in which odor-specific threshold differences to IVA were shown to be recessively inherited [18,19]. A subsequent linkage analysis of this phenotype found association with two distinct genomic loci, on mouse Chromosomes 4 and 6 [18]. Nevertheless, the genes underlying this sensory trait remain unknown. In the nematode Caenorhabditis elegans, on the other hand, mutations in a defined odorant receptor gene (*odr-10*) were shown to be responsible for specific response deficits to the odorant diacetyl [20].

Olfactory receptors (ORs) mediate the first step in odorant recognition [21], and their polymorphisms likely constitute the molecular basis for odorant-specific threshold variations. Of the 856 OR genes and pseudogenes dispersed throughout the entire human genome [22,23], a subclass of more than 60 OR loci appear particularly relevant, as they harbor damaging single nucleotide polymorphisms (SNPs) in their coding regions, leading them to segregate between an intact and a disrupted (pseudogenized) allele in the human population [24,25]. Each of these segregating pseudogenes (SPGs) is a natural knockout with a potential to underlie sensitivity variations towards certain chemosensory ligands. As a group, SPG loci harbor a remarkable combinatorial genetic diversity [24], perhaps one of the largest in the human genome [26].

To explore the possible involvement of OR SPGs in human olfactory threshold variation, we launched a genotype–phenotype association study of the underlying SNPs of 43 OR segregating loci and olfactory threshold measurements for four odorants in 377 individuals. We found a statistically significant association between the presence of a nonsense SNP within the coding region of the OR *OR11H7P* and detection threshold differences for the sweaty odorant IVA, suggesting a locus related to specific hyperosmia. This predicted OR–ligand relationship was validated by an in vitro expression assay [27]. Furthermore, we observed a significant concordance between odorant thresholds within individuals, likely governed by another inherent mechanism affecting general olfactory sensitivity. Our results suggest that the extensive olfactory threshold variation among humans is a complex trait, contributed to both by OR-specific variations and by potential inter-individual differences in downstream components in the olfactory signaling pathway.

## Results

### Human Olfactory Threshold Variation

**Figure 1:**
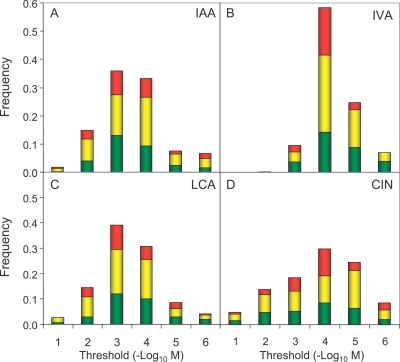
Olfactory Threshold Distributions Histograms of the measured olfactory thresholds for four odorants: IAA (A), IVA (B), LCA (C), and CIN (D). Odorants are expressed in molar (M) concentrations (in the oil solution) and were tested in 377 genotyped individuals, except CIN, which was tested in a randomly selected subsample of 200 participants. The data are presented for both genders pooled together. A threshold score of one indicates individuals who could not detect the highest possible odorant concentration (10^−2^ M). Red, yellow, and green, respectively, represent fractions out of the total sample for homozygote disrupted, heterozygote, and homozygote intact *OR11H7P* genotypes.

To study the possible genetic mechanism of human olfactory threshold variation, we measured detection thresholds of four odorants—isoamyl acetate (IAA), isovaleric acid (IVA), L-carvone (LCA), and cineole (CIN)—in 377 individuals. In accordance with previous observations [10], a broad Gaussian distribution was seen for all odorants, spanning the entire concentration range (10^−2^–10^−6^ M; Figure 1). The reproducibility of our threshold determination was indicated by a test–retest correlation (*R* = 0.73 ± 0.03, average of four odorants) in 82 randomly selected participants (Figure S1). Examination of other potential confounding factors revealed a noticeable difference between the thresholds of the two genders, with females exhibiting higher sensitivities towards all odorants (Table S1). In contrast, age, smoking habits, and ethnic origin did not show an effect.

### Genotype–Phenotype Association

**Figure 2:**
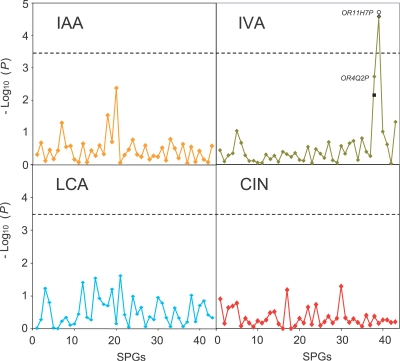
Genotype–Phenotype Association ANOVA *p*-values for comparison between the olfactory threshold distributions of participants with homozygous disrupted genotypes versus heterozygous and homozygous intact genotypes (1 df), using “gender” and “gender by genotype” as covariates. SPG loci are enumerated as in Table S2. The broken line indicates the statistically significant value of *p* = 0.05 after Bonferroni correction for 172 tests (four odorants × 43 SPGs). The two strongest *p*-values for IVA are for the genes marked in Figure 3. Using the individual's average threshold towards the four odorants as an additional covariate did not change the association signal with *OR11H7P* (open circle in IVA panel). The association signal with *OR4Q2P* is reduced after adjusting for the *OR11H7P* effect (solid square in IVA panel).

**Figure 3:**
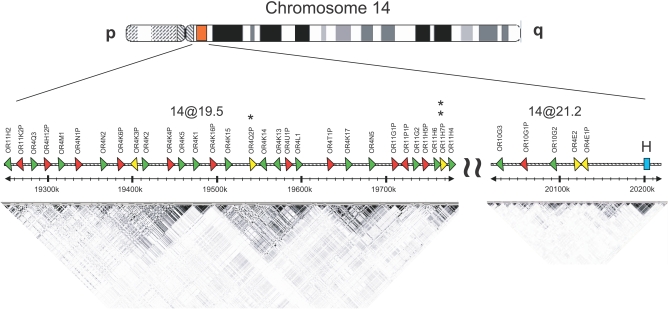
The Genomic Region Associated with IVA Hyperosmia OR genes are represented by red, green, and yellow triangles indicating pseudogenes, intact genes, and SPGs, respectively. The two SPGs showing the strongest association with IVA sensitivity are marked with single and double asterisks. A linkage disequilibrium plot of SNPs with minor allele frequency ≥ 5% in HapMap CEU [46] is depicted for this genomic region. The pairwise *r* ^2^ values are indicated on a gray scale, with black = 1 and white = 0. This region contains also the putative OR-specific *trans*-acting enhancer element (H) [47,48].

To test the possible involvement of OR SPGs in the observed odorant-specific olfactory threshold differences, we genotyped the underlying disrupting SNPs of 52 candidate OR SPGs (Table S2) in 377 individuals for whom genomic DNA was available. Of these, the genotypes of nine SNPs did not meet our quality assurance requirements (see Materials and Methods) and thus were removed from further scrutiny. The genotypes of the remaining 43 SNPs were further used for our genotype–phenotype analysis. Based on previous studies [11,13,18,19], we assumed Mendelian recessive inheritance of specific hyposmia, thus grouping together homozygous intact and heterozygous individuals. Comparison of the olfactory thresholds for individuals carrying different OR genotypes revealed a strong association signal between the genotyped SNP in *OR11H7P* and sensitivity to the odorant IVA (*F* =18.4, *p* = 2.29 × 10^−5^) (Figure 2). This association is explicitly revealed in the IVA threshold distribution (Figure 1B), in which participants homozygous for the disrupted allele (the pseudogene) are significantly depleted in the two highest olfactory sensitivity bins. That is, individuals with specific hyperosmia to IVA are more likely to be heterozygous or homozygous intact, than homozygous disrupted. This observation is consistent with the notion that individuals bearing no functional copy of *OR11H7P* would be incapable of detecting IVA at low concentrations. Interestingly, another SNP with an association signal with IVA detection (though not statistically significant at the α = 0.05 level) is in the *OR4Q2P* gene, which is situated ∼225 kb downstream of the *OR11H7P* gene and within the same OR genomic cluster, 14@19.5 (http://bioportal.weizmann.ac.il/HORDE) (Figure 3). The weak mutual linkage disequilibrium (*r* ^2^ = 0.13, *p* < 0.01) between these two polymorphic loci may contribute to the elevated association signal in the *OR4Q2P* gene as exemplified by the inferior statistical significance when both SNPs are fitted in the same analysis (Figure 2).

### In Vitro Functional Expression

**Figure 4:**
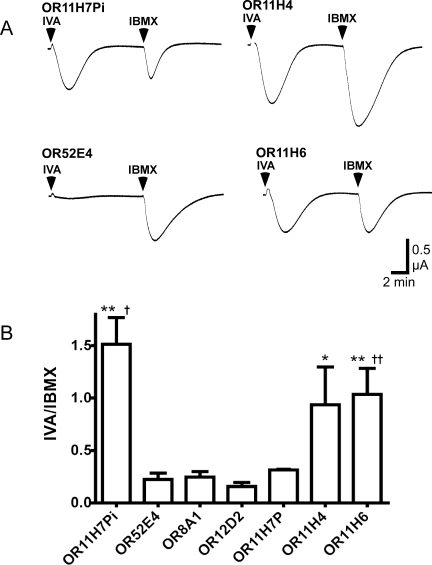
OR Responses to IVA (A) Representative current traces from *Xenopus* oocytes expressing the *OR11H7Pi*, *OR52E4*, *OR11H4*, or *OR11H6* receptor (see Materials and Methods) challenged with 15-s applications of 3 mM IVA and 1 mM IBMX. (B) Summary of the 4–12 recordings from oocytes expressing *OR11H7Pi*, *OR52E4*, *OR8A1*, *OR12D2*, *OR11H7P*, *OR11H4*, or *OR11H6*. Responses were normalized to the 1 mM IBMX response in the same oocyte and are presented as mean ± standard error of the mean. Significant differences from *OR52E4*, *OR8A1*, and *OR12D2*: *, *p* < 0.05; **, *p* < 0.01. Significant differences from *OR11H7P*: ^†^, *p* < 0.05; ^††^, *p* < 0.01.

Our genotype–phenotype analysis implies a functional relation between *OR11H7P* and the odorant IVA. To test this possibility, we coexpressed the intact allele of *OR11H7P* (*OR11H7Pi*) with human *GNAL* (*guanine nucleotide binding protein* [*G protein*], *alpha activating activity polypeptide, olfactory type*) and human *CFTR* (*cystic fibrosis transmembrane conductance regulator* [*ATP-binding cassette sub-family C*, *member 7*]) in *Xenopus* (frog) oocytes, and tested against various concentrations of IVA (from 30 μM to 10 mM; data not shown). *OR11H7Pi* responded to 3 mM IVA (Figure 4). In contrast, the uncorrected (pseudogenized) *OR11H7P* and three other randomly chosen intact human ORs (*OR52E4*, *OR8A1*, and *OR12D2*) did not yield specific responses to 3 mM IVA (Figure 4). Furthermore, we tested oocytes expressing an unrelated receptor (the rat α4β2 neuronal nicotinic receptor) and observed no responses to 3 mM IVA or to 1 mM 3-isobutyl-1-methylxanthine (IBMX) (data not shown). These results demonstrate that the response of *OR11H7Pi* to IVA is receptor specific. We also expressed and tested *OR11H4* and *OR11H6*, the two receptor genes that flank *OR11H7P* within the same linkage disequilibrium block (Figure 3). Similar to *OR11H7Pi*, both of these receptors responded to 3 mM IVA (Figure 4), but not to lower concentrations of IVA (data not shown). Notably, the average response signal of *OR11H7Pi* was higher than those of *OR11H4* and *OR11H6* (Figure 4B); however, these differences were not statistically significant. The three receptors that were activated by IVA did not respond to similar concentrations of IAA, LCA, or CIN (data not shown).

### Inter-Odorant Threshold Correlation

**Figure 5:**
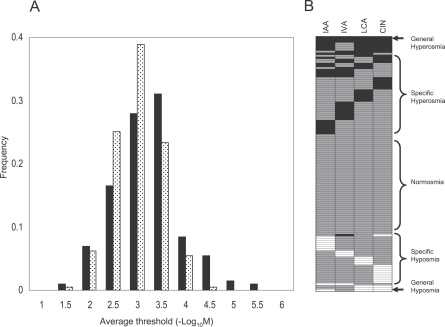
Excess of General Hyperosmia (A) Histogram of average olfactory thresholds (adjusted for gender). The average threshold values for the four odorants were calculated for the raw data (black bars) and for data generated by 1,000 permutations of the individual odorant thresholds (dotted bars). The significantly broader distribution of the original data as compared to the permutated data (ANOVA, *F* = 1.83, *p* = 1.68 × 10^−10^) indicates an excess of individuals with extreme threshold values, particularly in the hypersensitivity end of the distribution. (B) Combinations of odorant thresholds (adjusted for gender) for the four odorants. Shown are hyperosmia (lowest 10% of thresholds in the entire sample, black), normosmia (middle 80%, gray), and hyposmia (highest 10%, white). Individuals with similar threshold patterns are clustered together. For clarity, only 50 of the total of 123 normosmic individuals are shown. The probability of observing three individuals defined as generally hyperosmic (i.e., having hyperosmia to all four odorants) in this cohort is computed as ∼10^−12^.

A linear regression model reveals that the *OR11H7P* polymorphism explains ∼8% (*r* ^2^ = 0.08) of the IVA threshold variation in our data. Another ∼50% of this variation is attributed to intra-individual variance in our olfactory threshold measurements (Figure S1). Thus, ∼40% of the variation in human IVA sensitivity remains unexplained. We hypothesized that other mechanisms affecting the general olfactory sensitivity might contribute to the threshold variation towards IVA as well as to all other odorants. If true, this would be revealed as inter-odorant threshold concordances within individuals. Indeed, testing for such inter-odorant threshold correlations revealed statistically significant results (*p* < 0.01) for all odorant pairs (Figure S2). Notably, repeating the genotype–phenotype association analysis using the average olfactory threshold score as an additional covariate did not weaken the statistical significance of our results (see Figure 2). The effect of this general olfactory sensitivity is further demarcated by the observation that the average threshold distribution is significantly broader than the permuted one (*F* = 1.83, *p* = 1.68 × 10^−10^; Figure 5A). Interestingly, the difference between the original and permuted values was considerably larger in the lower values of the average thresholds, implying that general hyperosmia is more prevalent than general hyposmia in our sample. This is also evident in Figure 5B, which displays the variety of sensitivity phenotype combinations present. These observations imply that human sensitivity to IVA is determined by multiple factors that may have an odorant-specific or general olfactory effect.

## Discussion

The results presented here provide a link between genetic polymorphism in OR genes and phenotypic variation in human olfaction. Such genetic underpinning for the highly prevalent odorant-specific olfactory threshold differences in the human population has been previously suggested [9,28], but despite multiple reports of a genetic basis for these human phenotypes [11,13,16], the underlying genes remained unknown. Such a genotype–phenotype relationship has been found in the nematode C. elegans, in which an OR gene (*odr-10*) was shown to be necessary for specific responses to the odorant diacetyl [20]. This result implies that OR gene knockouts may underlie odor-specific threshold differences. Human olfactory SPGs in fact constitute natural knockout alleles, and the findings of the present study imply that they might underlie some of the phenotypic variation in human olfaction.

The genetic association between *OR11H7P* and IVA detection is further supported by in vitro functional analyses of this receptor that suggest a specific response of the intact protein to the odorant. The observation that OR11H4 and OR11H6 also respond to IVA is consistent with the notion that OR proteins from the same subfamily tend to bind similar odorants [29], and suggests that these three closely related OR genes likely confer IVA perception in humans. In view of that, specific hyperosmia for the sweaty odor of IVA would likely occur only in people who carry the intact allele of *OR11H7P* and hence possess enhanced detection capacity. Nevertheless, we cannot exclude the possibility that the observed association signal in this locus is a result of linkage disequilibrium to other genetic variations such as copy number polymorphisms [30], nonsynonymous SNPs in functionally crucial positions [25], or genetic alterations in regulatory sequences [31]. These could modify the expression levels of one or more receptor genes and could consequently underlie sensitivity differences towards the corresponding ligands of the encoded proteins.

The IVA concentration (3 mM) that elicits response from the three receptors from *OR11H7Pi* and its two other closely related ORs is three orders of magnitude higher than the threshold concentration detectable by individuals with hyperosmia to this odorant. Such differences are consistent with various observations in other studies. For example, olfactory thresholds in mice range from 10^−9^ to 10^−13^ M odorant concentration in the vapor phase [32,33], while the concentrations of odorants required for the activation of ORs in the isolated olfactory sensory neurons or in the heterologously expressed ORs were 10^−5^–10^−6^ M in the aqueous phase [34,35]. These discrepancies in sensitivity between in vivo responses to odorants in the vapor phase and the responses of dissociated olfactory sensory neurons to odorants in the aqueous phase have been attributed to the presence of olfactory mucus and the airflow in the nasal cavity present in in vivo experiments [36] and absent in the in vitro systems.

The human OR gene cluster 14@19.5 is in conserved synteny, and within the same “cluster in conservation” [37] as an OR cluster on mouse Chromosome 14. Notably, in mice, specific olfactory threshold variation to IVA was shown to be in genetic linkage to another OR locus (*Iva1*) on mouse Chromosome 4 [18,38]. The entire OR cluster in this genomic region is deleted in human [37,38]. Thus, it is possible that two different OR gene loci harbor the genetic basis for IVA sensitivity in the two mammals.

The results presented here imply a locus related to specific hyperosmia. Similar enhanced specific olfactory sensitivities have been reported only rarely, although support for a genetic basis for general hyperosmia is found in mice deficient for *Neu1* [39] and for the *Kcna3* genes [40]. A case of specific hyperosmia, for an unknown urinary metabolite of asparagus, was also attributed to genetic polymorphism in humans [15]; however, the underlying genetic locus has not been identified.

This study also highlights the potential phenotypic impact of often-neglected genomic loci—namely, pseudogenes. This is because most eukaryotic mRNA molecules that contain premature translation termination codons are either targeted for rapid degradation by a nonsense-mediated decay mechanism [41] or likely produce truncated nonfunctional protein. Thus, genomic loci annotated as pseudogenes are considered as bearing no functional consequence on the phenotype. Here we demonstrate that an OR annotated as a pseudogene in the public databases may still have a functional allele in the human population and consequently result in a discernible phenotype.

Genetic polymorphisms in OR genes likely explain only some of the remarkable threshold variation of human individuals towards particular odorants. Other potential contributing factors are intra-individual fluctuation [6] and environmental factors [14,15,42,43]. The observation of significant inter-odorant threshold correlation within individuals hints at the existence of a common mechanism that affects an individual's overall olfactory sensitivity. This is consistent with the observations of Cain and Gent [6], who postulated that such a mechanism may dominate the outcome of odorant-specific threshold measurements. While this effect could be related to differences in environmental exposures, another appealing possibility is the involvement of other genetic polymorphisms associated with variations in overall olfactory perception. The observation that the association signal between *OR11H7P* and IVA sensitivity did not change when the general olfactory effect was introduced into the statistical model implies that genetic polymorphisms in OR genes may dictate sensitivity towards particular odorants irrespective of other factors affecting overall olfactory sensitivity. Thus, both hyperosmia and hyposmia, the two extremes of human olfactory threshold distribution, could be driven by both receptor-specific mechanisms and additional elements common to all olfactory sensory neurons either in the olfactory transduction pathway or in processes related to olfactory cognition in the central nervous system. Further research endeavors in this direction would help to unravel additional pieces of the molecular basis of this fascinating complex sensory mechanism.

## Materials and Methods

### Participant recruitment.

This study was approved by the Institutional Review Board (Helsinki Committee) of Meir Hospital in Kfar Saba, Israel. Human participants (unrelated, randomly selected individuals) were recruited in collaboration with the Israeli Blood Bank (Tel Hashomer) within their blood-drawing sessions. Participants included 197 females and 246 males, aged 18–48 y, originating from three major Jewish ethnicities: 329 Ashkenazi, 61 Sephardic, and 53 Ashkenazi–Sephardic admixtures. Every participant signed an informed consent form and filled in a detailed questionnaire in order to exclude neurological impairments, nose injuries, and other conditions with potential effect on human olfactory acuity. Age, gender, ethnic origin, and smoking habits were also recorded.

### Olfactory threshold measurements.

Odorants, dissolved in light white mineral oil (Sigma) at five 10-fold dilutions between 10^−2^ M and 10^−6^ M were presented in Sniffin' Sticks kits [44]. These were replaced every 3 mo to reduce contamination and odorant evaporation. The odorants used were IAA (98%, Aldrich), IVA (99%, Sigma-Aldrich), LCA (97%, Aldrich), and CIN (≥98.5%, Fluka). Detection thresholds were determined using an ascending staircase three-way forced choice procedure [10]. Odor presentation by cap removal was for ∼3 s and presentation was ∼2 cm in front of both nostrils. A single failure led to the next higher concentration, and detection threshold was the concentration showing four successive correct identifications, assuring a low false positive rate (∼0.01). To attain reduction of false negative detection, the immediate subthreshold concentration was retested and claimed threshold if successful. Inter-trial intervals were >20 s, and participants were not given feedback on their performance during the test. All olfactory tests were conducted in a well-ventilated, temperature-controlled, odorless room. A second olfactory measurement was performed within 1–4 wk after the first one for 82 randomly selected individuals to assess test–retest reproducibility.

### SNP selection and genotyping.

Genomic DNA was extracted from 10 ml of peripheral blood using a DNA Isolation Kit for Mammalian Blood (Roche). The DNA concentrations were measured using the Genius spectrophotometer at 260 nm (Tecan) and subsequently normalized to 2.5 ng/μl. Aliquots of 2 μl were distributed in 384 microtiter plates by the Biomek 2000 laboratory automation workstation (Beckman).

We employed a candidate gene/SNP approach aimed at detecting the causative genetic polymorphism of human odor-specific threshold variation. Therefore, the underlying SNPs of 52 nonsense and missense SPGs [24,25], SNPs with obvious potential causative effect, were considered for the genotyping effort of this study. SNP genotypes were assessed by the matrix-assisted laser desorption/ionization–time of flight (MALDI-TOF) mass spectrometry technology of Sequenom [45]. This procedure involved multiplexes of 10–24 SNP reactions, automatically designed by the SpectroDESIGNER algorithm (Sequenom) and validated for specific genomic amplification using UCSC In-Silico PCR (http://genome.ucsc.edu/cgi-bin/hgPcr). SNP genotyping was performed twice, and inconsistencies were removed from further analyses. Overall, high genotyping efficiency (98% ± 3%) was achieved for the 52 polymorphic loci. SNP allele frequencies were tested for deviations from Hardy–Weinberg equilibrium. This test may indicate SNPs with errors in their genotyping process. Consequently, five SNPs were removed from further analysis (Table S2). Four additional SNPs with low minor allele frequency (<0.05; Table S2) were also not included in our association analyses because of the lack of statistical power to detect association even if existed. Thus, a total of 43 polymorphic loci remained for the genotype–phenotype association analysis.

### OR functional expression.

In vitro functional expressions of ORs were carried out as previously described [27]. OR coding regions were amplified from human genomic DNA by PCR using specific primers (BD Biosciences/Clontech), subcloned into a pCI vector (Promega) containing the 20-amino-acid N-terminal sequence of human rhodopsin (to aid in functional expression in this system), and confirmed by sequencing. The disrupting stop codon (TAG) (nucleotide thymine at position 679) of *OR11H7P* was corrected to a CAG codon for glutamine (as in the intact allele) using the QuickChange II site-directed mutagenesis kit (Stratagene), and verified by sequencing. Each Xenopus laevis oocyte was injected with 25 ng of the receptor cRNA, together with 10 ng of human *GNAL* cRNA and 1 ng of human *CFTR* cRNA. Currents, in response to IVA application, were measured under two-electrode voltage clamp. Results are reported as the ratio of current amplitudes elicited by the odorant and the phosphodiesterase inhibitor IBMX. Statistical analysis of functional data was done using one-way ANOVA, followed by Dunnett's post-test. X. laevis frogs were purchased from Nasco. The care and use of X. laevis frogs was approved by the University of Miami Animal Research Committee. Detailed methods for the preparation of oocytes have been previously described [27].

### Statistical analyses.

Analysis of variance (ANOVA) was used to test the potential effect of gender, ethnic origin (Ashkenazi Jewish, Sephardic Jewish, Ashkenazi–Sephardic Jewish admixture, or other), and smoking habits (>20 cigarettes/day, 11–20 cigarettes/day, 1–10 cigarettes/day, or nonsmoking) on odorant thresholds. Pearson correlation was used to assess the relationship between olfactory threshold and age of the participants. We used ANOVA to test for genotype–phenotype associations by comparing the average olfactory threshold of individuals with different SPG genotypes. For that, we assumed a recessive mode of inheritance (i.e., combining the heterozygous and homozygous intact genotypes) and adding “gender” and “gender by genotype” as other covariates. The recessive hypothesis was explicitly confirmed (*F* = 1.43, *p* = 0.24) by calculating the reduced fit between a 2-df test (additive) and 1-df test (recessive) using a likelihood ratio test. The interaction term “gender by genotype” did not have a significant effect (*F* = 0.17, *p* = 0.68 for “*OR11H7P* × gender”), therefore justifying the pooling of sexes in the analyses. The statistical analyses were carried out using the statistical toolbox of Matlab.

## Supporting Information

Figure S1 Test–Retest Reproducibility The first and second thresholds (in –log_10_ molar concentrations) of 82 individuals are plotted for the four odorants. Circle areas are proportional to the number of participants with similar scores. Test–retest reproducibility is demonstrated by the high correlation coefficient scores.
(23 KB PDF)

Figure S2 Inter-Odorant Threshold Correlations The adjusted thresholds (due to the gender effect) of individuals are plotted for all possible odorant pairs. Modest correlation coefficients (0.12 ≤ *R* ≤ 0.5, *p* < 0.01) in the different panels imply that a common denominator has a moderate effect on olfactory sensitivity towards all odorants.
(43 KB PDF)

Table S1 Olfactory Threshold Confounding Factors ANOVA (*F*) statistics and their corresponding *p*-values are given for the effect of gender, smoking, and ethnic origin on olfactory threshold variation for the different odorants. Pearson correlation coefficients (*R*) and their *p*-values indicate the effect of age on odorant thresholds.
(35 KB DOC)

Table S2 OR SPG Information OR SPGs are ordered according to their genomic location. The four genes with minor allele frequency below 0.05 (marked with a number sign) and the five genes with genotypes deviating significantly from Hardy–Weinberg (*p* < 0.05, Chi-square test; marked with an asterisk) were not included in the genotype–phenotype analyses.
(128 KB DOC)

## Abbreviations

ANOVA: analysis of variance
CIN: cineole
IAA: isoamyl acetate
IBMX: 3-isobutyl-1-methylxanthine
IVA: isovaleric acid
LCA: L-carvone
OR: olfactory receptor
SNP: single nucleotide polymorphism
SPG: segregating pseudogene
