# Supplementary material for: Genetic Elucidation of Human Hyperosmia to Isovaleric Acid
Source: PLoS Biol. 2007 Oct 30;5(11):e284. doi: 10.1371/journal.pbio.0050284 (PMC2043052; doi:10.1371/journal.pbio.0050284)
Supplement: Supplementary file 1 [file pbio.0050284.sg001.pdf]

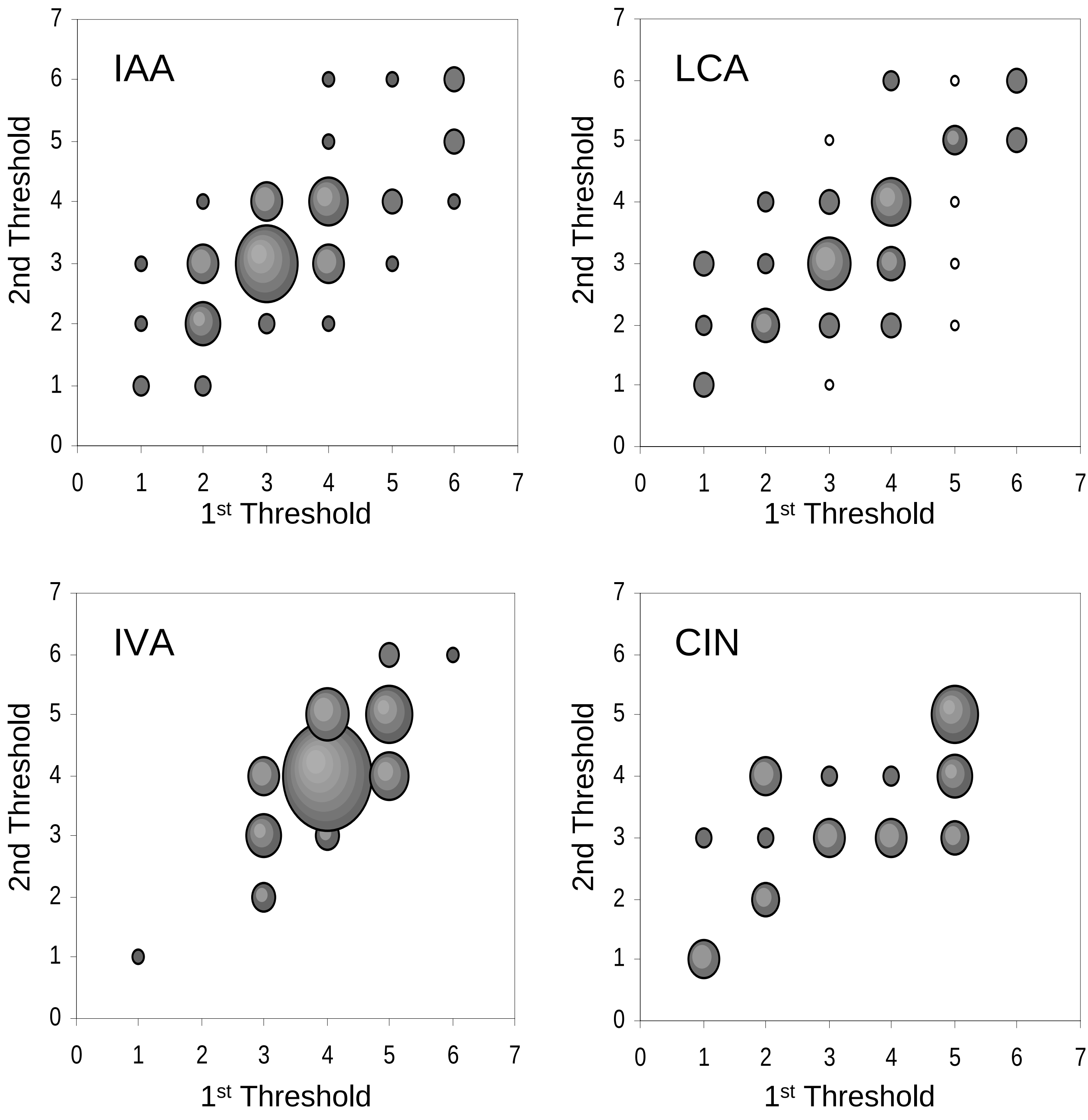
IAA
LCA
IVA
CIN
2nd Threshold
1st Threshold
0
1
2
3
4
5
6
7
