# Supplementary material for: Genetic Elucidation of Human Hyperosmia to Isovaleric Acid
Source: PLoS Biol. 2007 Oct 30;5(11):e284. doi: 10.1371/journal.pbio.0050284 (PMC2043052; doi:10.1371/journal.pbio.0050284)
Supplement: Supplementary file 2 [file pbio.0050284.sg002.pdf]

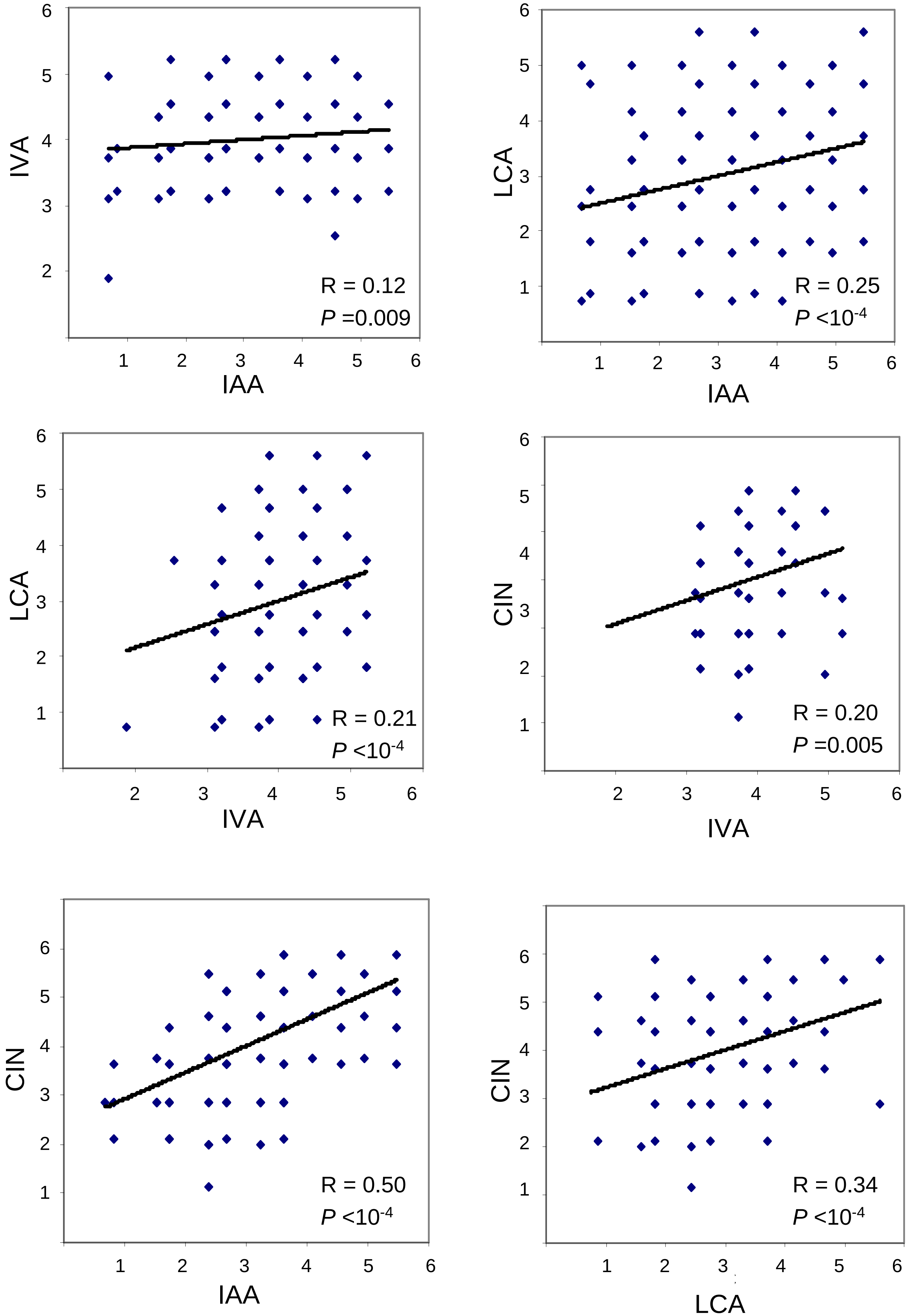
IVA
IAA
R = 0.12
P =0.009
LCA
IAA
R = 0.25
$P < 10^{-4}$
LCA
IVA
R = 0.21
$P < 10^{-4}$
CIN
IVA
R = 0.20
P =0.005
CIN
IAA
R = 0.50
$P < 10^{-4}$
CIN
LCA
R = 0.34
$P < 10^{-4}$
