# Supplementary material for: Genetic Elucidation of Human Hyperosmia to Isovaleric Acid
Source: PLoS Biol. 2007 Oct 30;5(11):e284. doi: 10.1371/journal.pbio.0050284 (PMC2043052; doi:10.1371/journal.pbio.0050284)
Supplement: Supplementary file 3 [file pbio.0050284.st001.doc]

|  | **IAA** | **IVA** | **LCA** | **CIN** |
| --- | --- | --- | --- | --- |
| **Gender** | **F = 10.18**  ***P* < 0.01** | **F = 7.71**  ***P* < 0.01** | **F = 7.31**  ***P < 0.01*** | F = 13.39  *P* < 0.01 |
| **Smoking** | **F = 0.4**  ***P* = 0.85** | **F = 0.51**  ***P* = 0.77** | **F = 1.1**  ***P* = 0.17** | F = 0.35  *P* = 0.88 |
| **Origin** | **F = 0.32**  ***P* = 0.26** | **F = 0.9**  ***P* = 0.11** | **F = 0.66**  ***P* = 0.65** | F = 0.48  *P* = 0.79 |
| **Age** | R = -0.08  P = 0.10 | R = -0.003  P = 0.95 | R = -0.08  P = 0.10 | R = -0.05  P = 0.50 |

**Table S1**

**Olfactory threshold confounding factors**.

ANOVA (F) statistics and their corresponding *P*-values of are given for the effect of Gender, Smoking and Ethnic origin on olfactory thresholds variation of the different odorants. Pearson correlation coefficients (R) and their *P*-values indicate the effect of age on odorant thresholds.
