# Supplementary material for: Genetic Elucidation of Human Hyperosmia to Isovaleric Acid
Source: PLoS Biol. 2007 Oct 30;5(11):e284. doi: 10.1371/journal.pbio.0050284 (PMC2043052; doi:10.1371/journal.pbio.0050284)
Supplement: Supplementary file 4 [file pbio.0050284.st002.doc]

| **No.** | **Gene information** | | **Polymorphism information** | | | | **Genotyping frequencies** | | | **Allele frequencies** | | **Hardy-Weinberg equilibrium** |
| --- | --- | --- | --- | --- | --- | --- | --- | --- | --- | --- | --- | --- |
|  | **Name** | **Genomic**  **location** | **Position** | **Type** | **Intact**  **allele** | **Pseudogene**  **allele** | **Homozygote**  **Intact** | **Heterozygote** | **Homozygote**  **pseudogene** | **Intact**  **allele** | **Pseudogene**  **allele** | ***P* of Chi square test** |
| 1 | OR10X1 | 1@155.4 | 147 | Stop | G | A | 0.29 | 0.508 | 0.202 | 0.544 | 0.456 | 0.899 |
| 2 | OR10J4P | 1@156.2 | 593 | Del(1) | T | - | 0.867 | 0.13 | 0.003 | 0.932 | 0.068 | 0.838 |
| 3 | OR2L8 | 1@244.6 | 650 | Y226C | A | G | 0.005 | 0.186 | 0.808 | 0.098 | 0.901 | 0.645 |
| 4 | OR5H6 | 3@99.5 | 385 | P137A | C | G | 0.419 | 0.448 | 0.133 | 0.643 | 0.357 | 0.896 |
| 5 | OR5H6 | 3@99.5 | 535 | C187R | T | C | 0.162 | 0.411 | 0.426 | 0.3675 | 0.6315 | 0.109 |
| 6 | OR5AC1P | 3@99.5 | 257 | Del(1) | T | - | 0.336 | 0.53 | 0.134 | 0.601 | 0.399 | 0.131 |
| 7 | OR12D1P | 6@29.4 | 556-572 | Del(16) | GCCTGTGGGAACACTG | - | 0.117 | 0.473 | 0.41 | 0.3535 | 0.6465 | 0.79 |
| 8 | OR12D2 | 6@29.4 | 359 | R130L | G | T | 0.184 | 0.524 | 0.291 | 0.446 | 0.553 | 0.507 |
| 9 | OR2J1P | 6@29.4 | 574 | Stop | C | T | 0.296 | 0.494 | 0.209 | 0.543 | 0.456 | 0.998 |
| 10 | # OR10C1 | 6@29.4 | 163 | Stop | C | T | 0.935 | 0.065 | 0 | 0.9675 | 0.0325 | 0.812 |
| 11 | * OR10AC1P | 7@142.7 | 267 | In(1) | - | G | 0.523 | 0.341 | 0.136 | 0.6935 | 0.3065 | 0.002 |
| 12 | OR2F1 | 7@143.3 | 364 | R130C | C | T | 0.872 | 0.118 | 0.011 | 0.931 | 0.07 | 0.215 |
| 13 | OR1B1 | 9@122.5 | 574 | Stop | C | T | 0.427 | 0.462 | 0.111 | 0.658 | 0.342 | 0.887 |
| 14 | OR13C7P | 9@35.9 | 435-436 | In(2) | - | AA | 0.034 | 0.247 | 0.719 | 0.1575 | 0.8425 | 0.376 |
| 15 | OR8B4 | 11@123.6 | 532 | C187R | T | C | 0.52 | 0.379 | 0.101 | 0.7095 | 0.2905 | 0.302 |
| 16 | OR8D2 | 11@123.6 | 365 | R130H | G | A | 0.592 | 0.366 | 0.042 | 0.775 | 0.225 | 0.647 |
| 17 | OR8G1 | 11@123.6 | 777 | Stop | C | G | 0.227 | 0.539 | 0.235 | 0.4965 | 0.5045 | 0.325 |
| 18 | OR4X2 | 11@48.4 | 81 | Stop | C | G | 0.807 | 0.19 | 0.003 | 0.902 | 0.098 | 0.323 |
| 19 | OR51J1 | 11@5.0 | 299 | C105Y | G | A | 0.743 | 0.225 | 0.032 | 0.8555 | 0.1445 | 0.229 |
| 20 | OR52B4 | 11@5.0 | 119 | Del(1) | C | - | 0.444 | 0.46 | 0.097 | 0.674 | 0.327 | 0.686 |
| 21 | * OR51F1 | 11@5.0 | 274 | Del(1) | C | - | 0.422 | 0.408 | 0.17 | 0.626 | 0.374 | 0.046 |
| 22 | OR52E1P | 11@5.0 | 778 | Del(1) | C | - | 0.269 | 0.476 | 0.255 | 0.507 | 0.493 | 0.652 |
| 23 | OR52Z1P | 11@5.0 | 22 | Del(1) | G | - | 0.102 | 0.404 | 0.493 | 0.304 | 0.695 | 0.68 |
| 24 | OR52R1 | 11@5.0 | 386 | I134T | C | T | 0.239 | 0.515 | 0.247 | 0.4965 | 0.5045 | 0.851 |
| 25 | OR51B2 | 11@5.0 | 358 | R130C | C | T | 0.814 | 0.175 | 0.011 | 0.9015 | 0.0985 | 0.977 |
| 26 | OR51G1 | 11@5.0 | 371 | R130H | G | A | 0.707 | 0.253 | 0.04 | 0.8335 | 0.1665 | 0.229 |
| 27 | OR52H1 | 11@5.0 | 371 | R130H | G | A | 0.663 | 0.31 | 0.027 | 0.818 | 0.182 | 0.714 |
| 28 | OR51Q1 | 11@5.0 | 706 | Stop | C | T | 0.285 | 0.495 | 0.221 | 0.5325 | 0.4685 | 0.992 |
| 29 | OR52D1 | 11@5.0 | 662 | Y226F | A | T | 0.684 | 0.279 | 0.037 | 0.8235 | 0.1765 | 0.73 |
| 30 | OR52L1 | 11@5.9 | 373 | R130C | C | T | 0.547 | 0.373 | 0.08 | 0.7335 | 0.2665 | 0.679 |
| 31 | OR52N4 | 11@5.9 | 514 | Stop | A | T | 0.62 | 0.335 | 0.045 | 0.7875 | 0.2125 | 1 |
| 32 | # OR5R1 | 11@55.6 | 362 | D129R | A | G | 0.928 | 0.067 | 0.005 | 0.9615 | 0.0385 | 0.137 |
| 33 | OR5R1 | 11@55.6 | 364 | R130C | C | T | 0.597 | 0.357 | 0.046 | 0.7755 | 0.2245 | 0.89 |
| 34 | OR5G3P | 11@55.6 | 343 | Del(1) | C | - | 0.333 | 0.44 | 0.227 | 0.553 | 0.447 | 0.104 |
| 35 | OR5AL1P | 11@55.6 | 468-469 | Del(2) | CT | - | 0.851 | 0.146 | 0.003 | 0.924 | 0.076 | 0.694 |
| 36 | OR5L1 | 11@55.6 | 858 | P298S | C | T | 0.006 | 0.133 | 0.861 | 0.0725 | 0.9275 | 0.978 |
| 37 | OR8K3 | 11@55.6 | 365 | R130L | G | T | 0.363 | 0.461 | 0.176 | 0.5935 | 0.4065 | 0.695 |
| 38 | # OR8J2P | 11@55.6 | 190 | Stop | C | T | 0.008 | 0.08 | 0.912 | 0.048 | 0.952 | 0.054 |
| 39 | OR5AR1 | 11@55.6 | 55 | Stop | C | T | 0.109 | 0.43 | 0.462 | 0.324 | 0.677 | 0.938 |
| 40 | OR6Q1 | 11@57.7 | 685 | Del(1) | C | - | 0.719 | 0.26 | 0.021 | 0.849 | 0.151 | 0.97 |
| 41 | OR1S1 | 11@57.7 | 365 | R130H | G | A | 0.214 | 0.466 | 0.32 | 0.447 | 0.553 | 0.547 |
| 42 | OR10A6 | 11@7.8 | 860 | P298L | C | T | 0.597 | 0.345 | 0.058 | 0.7695 | 0.2305 | 0.856 |
| 43 | * OR4K3P | 14@19.5 | 622 | Del(1) | C | - | 0.608 | 0.378 | 0.014 | 0.797 | 0.203 | 0.006 |
| 44 | OR4Q2P | 14@19.5 | 531 | In(1) | - | T | 0.152 | 0.423 | 0.425 | 0.3635 | 0.6365 | 0.263 |
| 45 | OR11H7P | 14@19.5 | 679 | Stop | C | T | 0.308 | 0.475 | 0.217 | 0.5455 | 0.4545 | 0.708 |
| 46 | OR4E1P | 14@21.2 | 584-585 | In(2) | - | AC | 0.605 | 0.345 | 0.05 | 0.7775 | 0.2225 | 0.996 |
| 47 | OR4E2 | 14@21.2 | 352 | M126V | A | G | 0.604 | 0.348 | 0.048 | 0.778 | 0.222 | 0.987 |
| 48 | OR6J1 | 14@22.2 | 362 | R130H | G | A | 0.745 | 0.244 | 0.011 | 0.867 | 0.133 | 0.513 |
| 49 | * OR1A1 | 17@3.1 | 853 | P298S | C | T | 0.308 | 0.435 | 0.257 | 0.5255 | 0.4745 | 0.046 |
| 50 | # OR7C2 | 17@3.1 | 365 | R130H | G | A | 0.92 | 0.077 | 0.003 | 0.9585 | 0.0415 | 0.894 |
| 51 | * OR3A1 | 17@3.1 | 374 | R130Q | G | A | 0.313 | 0.372 | 0.315 | 0.499 | 0.501 | 0 |
| 52 | OR1P1P | 17@3.1 | 523 | Stop | A | T | 0.014 | 0.252 | 0.734 | 0.14 | 0.86 | 0.651 |

**Table S2**

OR SPGs information.

OR SPGs are ordered according to their genomic location. The four genes with minor allele frequency below 0.05 (Marked with #) and five genes with genotypes deviating significantly from Hardy-Weinberg (P <0.05 of *Chi-square* test, marked with *) were not included in the phenotype-genotype analyses.
